# Modelling the global constraints of temperature on transmission of *Plasmodium falciparum *and *P. vivax *

**DOI:** 10.1186/1756-3305-4-92

**Published:** 2011-05-26

**Authors:** Peter W Gething, Thomas P Van Boeckel, David L Smith, Carlos A Guerra, Anand P Patil, Robert W Snow, Simon I Hay

**Affiliations:** 1 Spatial Ecology and Epidemiology Group, Tinbergen Building, Department of Zoology, University of Oxford, South Parks Road, Oxford OX1 3PS, UK; 2 Biological Control and Spatial Ecology, Université Libre de Bruxelles, CP160/12, Avenue FD Roosevelt 50, B-1050 Brussels, Belgium; 3 Emerging Pathogens Institute, University of Florida, Gainesville, Florida 32610, USA; 4 Department of Biology, University of Florida, Gainesville, Florida 32610, USA; 5 Malaria Public Health and Epidemiology Group, Centre for Geographic Medicine, KEMRI - University of Oxford - Wellcome Trust Collaborative Programme, Nairobi, Kenya; 6 Centre for Tropical Medicine, Nuffield Department of Clinical Medicine, University of Oxford, CCVTM, Oxford, OX3 9DS, UK

## Abstract

**Background:**

Temperature is a key determinant of environmental suitability for transmission of human malaria, modulating endemicity in some regions and preventing transmission in others. The spatial modelling of malaria endemicity has become increasingly sophisticated and is now central to the global scale planning, implementation, and monitoring of disease control and regional efforts towards elimination, but existing efforts to model the constraints of temperature on the malaria landscape at these scales have been simplistic. Here, we define an analytical framework to model these constraints appropriately at fine spatial and temporal resolutions, providing a detailed dynamic description that can enhance large scale malaria cartography as a decision-support tool in public health.

**Results:**

We defined a dynamic biological model that incorporated the principal mechanisms of temperature dependency in the malaria transmission cycle and used it with fine spatial and temporal resolution temperature data to evaluate time-series of temperature suitability for transmission of *Plasmodium falciparum *and *P. vivax *throughout an average year, quantified using an index proportional to the basic reproductive number. Time-series were calculated for all 1 km resolution land pixels globally and were summarised to create high-resolution maps for each species delineating those regions where temperature precludes transmission throughout the year. Within suitable zones we mapped for each pixel the number of days in which transmission is possible and an integrated measure of the intensity of suitability across the year. The detailed evaluation of temporal suitability dynamics provided by the model is visualised in a series of accompanying animations.

**Conclusions:**

These modelled products, made available freely in the public domain, can support the refined delineation of populations at risk; enhance endemicity mapping by offering a detailed, dynamic, and biologically driven alternative to the ubiquitous empirical incorporation of raw temperature data in geospatial models; and provide a rich spatial and temporal platform for future biological modelling studies.

## Background

Amongst many natural and anthropogenic factors, ambient temperature plays a key role in determining the suitability of local environments for transmission of human malaria. At the extremes, temperature regimes constrain the geographical extent of the disease and, within this envelope, contribute to determining its intensity. These constraints are temporally dynamic, with fluctuations in transmission suitability and intensity driven by seasonal and inter-annual temperature cycles. The importance of temperature as an environmental determinant of malaria endemicity arises from a series of effects on the life cycles of the *Plasmodium *parasite and *Anopheles *mosquito vector, and a lineage of theoretical and experimental studies stretching back some 80 years [1] has sought to understand and model these mechanisms.

The same period has seen parallel developments in the methods and scope of malaria cartography. Early efforts to map the extent and severity of human malaria worldwide, heavily reliant on subjective judgment and manual data extrapolation [2], have been supplanted in recent years by a new generation of evidence-based global maps [3-6] generated systematically using large assemblies of malariometric data [7] and modern spatial and space-time statistical modelling methods [8]. These contemporary maps have, in turn, facilitated assessment of a wide range of measures central to the planning, implementation, and monitoring of disease control including the evaluation of global populations at risk [3,5,6], clinical burden [9], equity in international funding for disease control [10,11], and relative national feasibility of elimination [12]. Given the importance of temperature in shaping the landscape of malaria, an understanding and quantitative representation of its effects forms an essential component of effective malaria cartography as a decision support tool in public health.

A variety of approaches have been developed for incorporating temperature information in national, regional and global scale malaria cartographic models, but each suffers limitations. Spatial statistical models have tended to incorporate temperature data directly as a mapped covariate, using empirically defined relationships with observed infection prevalence to inform prediction of the latter quantity across space (for example [4,13-23]). This approach is limited because the mechanisms of temperature dependence on vector and parasite dynamics are highly non-linear and raw temperature values are likely to be only indirectly linked to observed transmission intensity. An empirical model inevitably captures this complexity imperfectly and does not leverage the substantial body of theoretical understanding amassed from biological modelling studies. More practically, empirical evaluation of temperature effects at the limits of transmission suitability is hampered by the dearth of reliable prevalence data from these marginal zones [4], making the delineation of areas at risk problematic.

The natural counterpart to an empirical handling of temperature is a deterministic approach that uses mechanistic models to capture known biological mechanisms of temperature dependence. At the continental or global scale, such models have been used to map various indices of environmental suitability, either to support endemicity mapping directly [5,6], or as part of efforts to model contemporary or potential future climatic suitability [24-32]. A common limitation of these studies, however, is the simplistic handling of temporal dynamics. Temperature effects are often evaluated at a series of fixed points in an annual cycle, for example based on mean monthly temperature values and therefore providing twelve snapshots of the theoretical suitability of conditions for transmission under assumptions of constant temperatures. In reality, vectors and parasites experience a continuously changing temperature regime throughout their lifespan and the resulting dynamics cannot be captured fully in a static analysis. More elaborate mechanisms for investigating the dynamic effects of temperature on vector and parasite populations have been developed in smaller scale or theoretical modelling studies [33-38]. These dynamic models are not only more realistic biologically, but potentially allow temporal changes in temperature-driven transmission suitability to be described in considerable detail across a year.

The aim of the current study is to provide new global maps describing the constraining effects of temperature on transmission of the two main malaria parasites of public health concern, *Plasmodium falciparum *and *P. vivax*. By defining a dynamic model in which the effects of continuously changing temperature regimes experienced by vector and parasite populations are incorporated, using high spatial resolution interpolated temperature data, and accounting for diurnal as well as seasonal temperature cycles, we present the first spatially and temporally detailed description of temperature constraints on transmission at the global scale.

## Methods

### Modelling framework

A natural analytical framework for considering the effects of temperature on malaria transmission intensity is provided by deterministic models for the disease's basic reproductive number, *R *_0 _, defined formally as the expected number of new cases arising in a naive population after one generation of the parasite from the introduction of a single infectious person [39-41]. These models parameterise malaria transmission in terms of characteristics of, and interactions between, human, vector, and parasite populations [42-46]. Those aspects of the transmission cycle affected by temperature are encapsulated in a component of *R *_0 _known as vectorial capacity [47], *V *, which defines the total number of subsequent infectious bites arising from a single person-day of exposure and is classically expressed as:(1)

where *m *is the number of mosquitoes per human, *a *is the human feeding rate, *p *is the daily vector survival rate, and *n *is the time required for sporogony, the maturation of parasites ingested by mosquitoes during human blood meals into the sporozoite life cycle stage infectious to humans. Expressing vector survival in terms of daily death rate, *g *where, *g *= -ln *p*, and holding constant the rate of adult mosquito recruitment, λ, relative to the human population so that, *m *= *λ*/*g*, vectorial capacity can be rewritten [48] as:(2)

Temperature can influence all of the terms in this equation. Temperature affects feeding rates, *a *for example, via effects on vector activity and blood meal digestion [49-51]. Larval ecology and, thus, adult recruitment, λ, are affected by temperatures found in aquatic habitats which play a role in modulating larval development rates and survival [33,34,52,53]. Other work has demonstrated how these factors alone can impose limits on habitat suitability for particular anopheline species [53]. Adult mosquito recruitment is, however, also driven by a myriad of other climatic and local environmental factors, in particular those associated with the often transitory availability of aquatic oviposition sites. Here we focus on the more pronounced and directly measurable effects of temperature on vectorial capacity: the interaction between vector lifespan, determined by, *g *and the duration of sporogony, *a*. Holding *a *and λ constant, then, we can modify equation (2) to obtain an expression as a function of temperature, *T*:(3)

Since *a *and λ are unknown, vectorial capacity cannot be evaluated directly, so we define instead an index of temperature suitability *Z(T) *that is linearly proportional to *V(T) *and therefore sufficient for exploring the relative, rather than absolute, effect of temperature on vectorial capacity and, thus, on *R *_0 _. The index *Z(T) *can be interpreted as a relative measure of the number of infectious mosquitoes supported in an environment with temperature *T*, given a constant emergence rate λ. All other things being equal, an environment with, say *Z(T)*, a value of 100 would support twice the vectorial capacity or, equivalently, require half as many vectors to support the same vectorial capacity as one with a *Z(T) *value of 50. Locations in which *Z(T) *is zero indicate that no vectors survive long enough to accumulate sufficient degree days for sporogony.

### Temperature effects on sporogony and vector survivorship

Drawing on a series of observational and modelling studies [54-56], and assuming no indirect influences of temperature such as effects on humidity or evapotranspiration [24,57], death rate as a function of temperature in degrees Celsius, *g(T)*, can be modelled as:(4)

The dependence of sporogony duration, *n*, on temperature is classically expressed using a simple temperature-sum model [40] in which sporogony occurs after a fixed number of degree-days, ϕ, over a minimum temperature threshold for development, *T*_min_:(5)

with widely used parameterisations from studies on *Anopheles maculipennis *[1,50] defining a degree-day requirement of ϕ = 105 and ϕ = 111 for *P. vivax *and *P. falciparum *respectively, and corresponding minimum temperatures for development of *T*_min_= 14.5°C and *T*_min_= 16°C.

### Dynamic model evaluation

In a static approach, that is, using fixed temperature values such as monthly means, equations (4) and (5) can be evaluated directly to obtain values for *g *and *n *which can, in turn, be used in equation (3) to calculate a relative value for vectorial capacity. Although widely used in modelling studies, this static approach cannot recreate the effects on parasite and vector cohorts of the continuously changing temperature regime they experience across their lifespan. In a real-world environment where temperature varies through time, equation (3) can be interpreted as combining three distinct temperature-dependent components of the transmission cycle, as follows. Firstly, the numerator describes the probability of vectors surviving the duration of sporogony and can be evaluated by integrating the survivorship curve defined by the temperature-dependent death rate, *g(T(τ))*, between the onset of sporogony (at time *τ *= *n*) and the completion of sporogony *n *days later (at time τ = 0):(6)

Under a continuously changing temperature regime, the simple expression for *n *described above (5) is insufficient and instead a value must be calculated that satisfies the following equation:(7)

Secondly, the first of the two *g(T) *terms in the denominator relates to vector death rates prior to becoming infected (*i.e*. before the onset of sporogony at time *τ *= *n*). The survivorship of vector cohorts during this period can be evaluated by a double integral that tracks death rates of successive vector cohorts between their emergence (at time *τ *= *n*-*t*) and the onset of sporogony (at time *τ *= *n*):(8)

Thirdly, the second of the two *g(T) *terms in the denominator relates to vector survivorship after becoming infectious, and can be evaluated by another double integral that sums the survivorship of extant vector cohorts following completion of sporogony (at time *τ *= 0), the period during which transmission to humans can occur:(9)

Combining these three terms, then, we obtain an expression for *Z(T) *indexed temporally to the point of infectiousness at *τ *= 0:(10)

### Model implementation with realistic temperature regimes

Temperature data were obtained from the WorldClim data resource [58] in the form of long-term (1950-2000) mean, minimum, and maximum interpolated climatology surfaces for each synoptic calendar month. Monthly values for each pixel were smoothed temporally with spline interpolation and a sinusoidal diurnal cycle added which has been shown to be an important modulator of temperature effects on transmission [35,59]. More detail on these data, the rationale for their use, and the pre-processing implemented to provide realistic annual temperature regimes is provided in Additional File 1.

The dynamic temperature suitability index described in equation (10) was evaluated separately for *P. falciparum *and *P. vivax *using a system of difference equations with a two hour time-step to provide time-series of *Z(T) *for every 1 × 1 km land pixel globally. Further details of the computational implementation are provided in Additional File 2. Because vector death rates were modelled only as a function of temperature, and not of age, proportional survival curves approached zero (*i.e*. complete mortality of a given cohort) asymptotically. In order to identify areas and times when local temperature regimes were likely to preclude any transmission, a maximum vector lifespan was imposed beyond which daily survival was set to zero. Vector lifespans were defined following the same rationale and systematic literature review presented previously [5]. In brief, a maximum longevity of 31 days was used because estimates of the lifespan of the main dominant vectors [29] indicate that more than 99% of anophelines die in less than this period, with an exception made for areas that support the longer-lived *Anopheles sergentii *and *An. superpictus *[60] as dominant vectors, in which 62 days was considered more appropriate [29].

### Summarising and mapping the temperature suitability index

*Z(T) *time-series values of zero indicated that the local temperature regime within a given pixel cannot support the presence of infectious vectors at that time point. This arises when no extant vectors have been able to accumulate sufficient degree days to support sporogony. By extension, time-series with only zero values were indicative of pixels permanently unable to support transmission and could be classified as risk-free locations. This situation arises when no time windows exist throughout the year when the extrinsic incubation period is less than maximum feasible vector lifespans. Every pixel was classified in this way as either suitable or unsuitable for transmission and the resulting binary values mapped for both *P. falciparum *and *P. vivax *to create two global grids delineating the absolute limits of temperature suitability for transmission of each species worldwide. As a measure of the annual duration of suitable conditions within each pixel, the total proportion of non-zero values throughout the year, expressed to the nearest whole day, was calculated for each pixel and plotted globally for each parasite. Regions with year-round suitability thus displayed pixel values of 365 whilst in seasonally suitable regions the value was smaller, and in perennially unsuitable regions the value was zero. Finally, as a summary of the degree of temperature suitability across the year for each parasite species, the area under the *Z(T) *time-series was calculated and plotted for each pixel. Since this integral provided a relative, rather than absolute, summary measure of annual temperature suitability, the arbitrary units were rescaled to lie between zero and one by dividing by the global maximum. To allow comparison between the two species' maps, the maximum global value for *P. vivax *was used to rescale both.

For each parasite species, the three maps described above displayed different summaries of temperature suitability across the year and allowed spatial comparison of relative suitability from place to place. To describe the information contained in the time-series at each pixel more completely, temporal disaggregation is required. This was achieved by generating 52 separate maps corresponding to the mid-point of each week of the year. Each map displayed the area under the *Z(T) *time-series for that week, rescaled to correspond to the annual integral by multiplying by 52. Animations were then generated for each species that displayed these weekly maps in sequence, allowing visualisation of the seasonal evolution of temperature suitability.

## Results

### Example time-series of temperature-dependent transmission suitability

**Figure 1:**
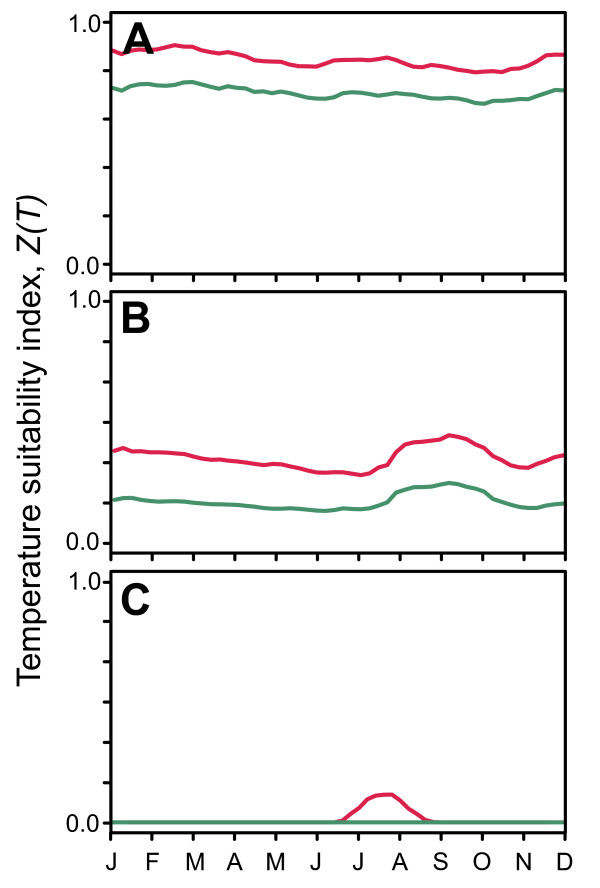
**Temperature suitability index time-series for three example pixels**. Each plot shows the *Z(T) *modelled value evaluated weekly across an average year for *P. falciparum *(green line) and *P. vivax *(red line) for individual pixels located in (A) Central Sumatra; (B) Western Kenya; (C) Central Afghanistan.

Figure 1 displays example time-series of the *Z(T) *temperature suitability index for both *P. falciparum *and *P. vivax *from three pixels located in parts of the malaria endemic world with very different temperature regimes. Plot A, taken from a pixel in central Sumatra illustrates consistently high values of *Z(T) *with almost no seasonal variation, indicative of an annual temperature regime that can support high relative values of vectorial capacity, for a given vector recruitment rate, year-round. Because both the minimum temperature and degree-day requirement for sporogony are lower for *P. vivax *than *P. falciparum *, temperature suitability will always be slightly higher for the former, and this is reflected in the two time-series. Plot B is from the fringe of the western Kenyan highlands. In comparison to the Sumatra pixel, the annual temperature regime here results in lower values of the suitability index, although temperature does not preclude transmission of either parasite at any point in the year, and a period of slightly elevated suitability is evident in August, September, and October. The most pronounced seasonality is displayed in Plot C, extracted from a pixel in central Afghanistan. Here, temperatures are too cold to support transmission of either parasite for the majority of the year with the exception of a distinct three month period in the summer during which *P. vivax *transmission becomes feasible, reflecting its lower sensitivity to cool temperatures. Time-series like these three examples, available for every 1 × 1 km pixel globally, provide a rich class of information about the seasonal dynamics of temperature-driven suitability, taking into account the annual and diurnal temperature regimes and their cumulative effects on local vector longevity and parasite development.

### Mapped indices of temperature-dependent transmission suitability

**Figure 2:**
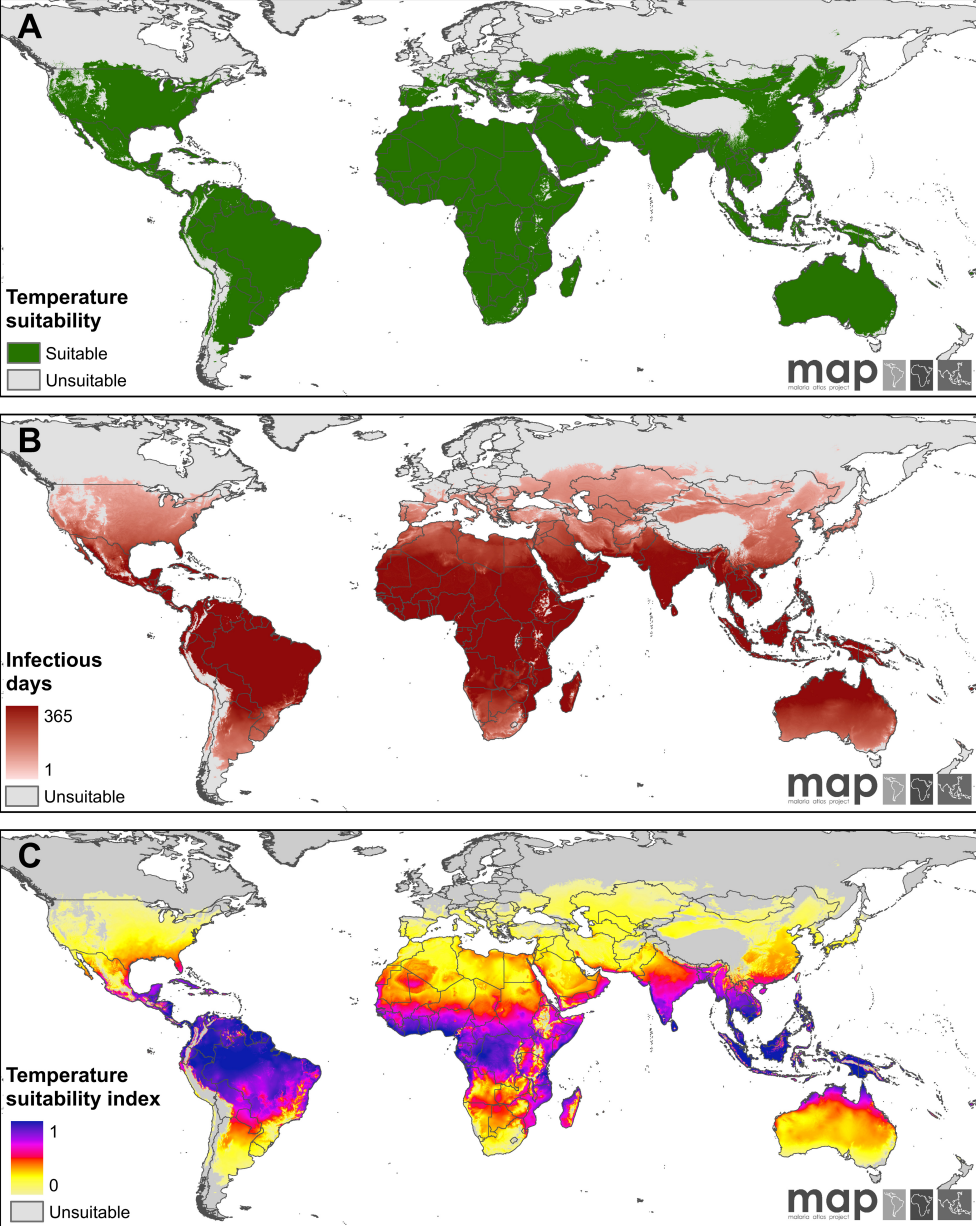
**Mapped outputs of the temperature suitability model for *P. falciparum***. (A) Modelled limits of temperature suitability for transmission of *P. falciparum*. Areas shaded grey are those in which the annual temperature regime would be unable to support infectious vectors at any point during an average year. (B) The number of days in an average year in which the annual temperature regime could support potentially infectious vectors. (C) The normalized *Z(T) *index of temperature suitability that incorporates not just the duration but also the degree of suitability across an average year. See text for a full explanation of all three metrics.

**Figure 3:**
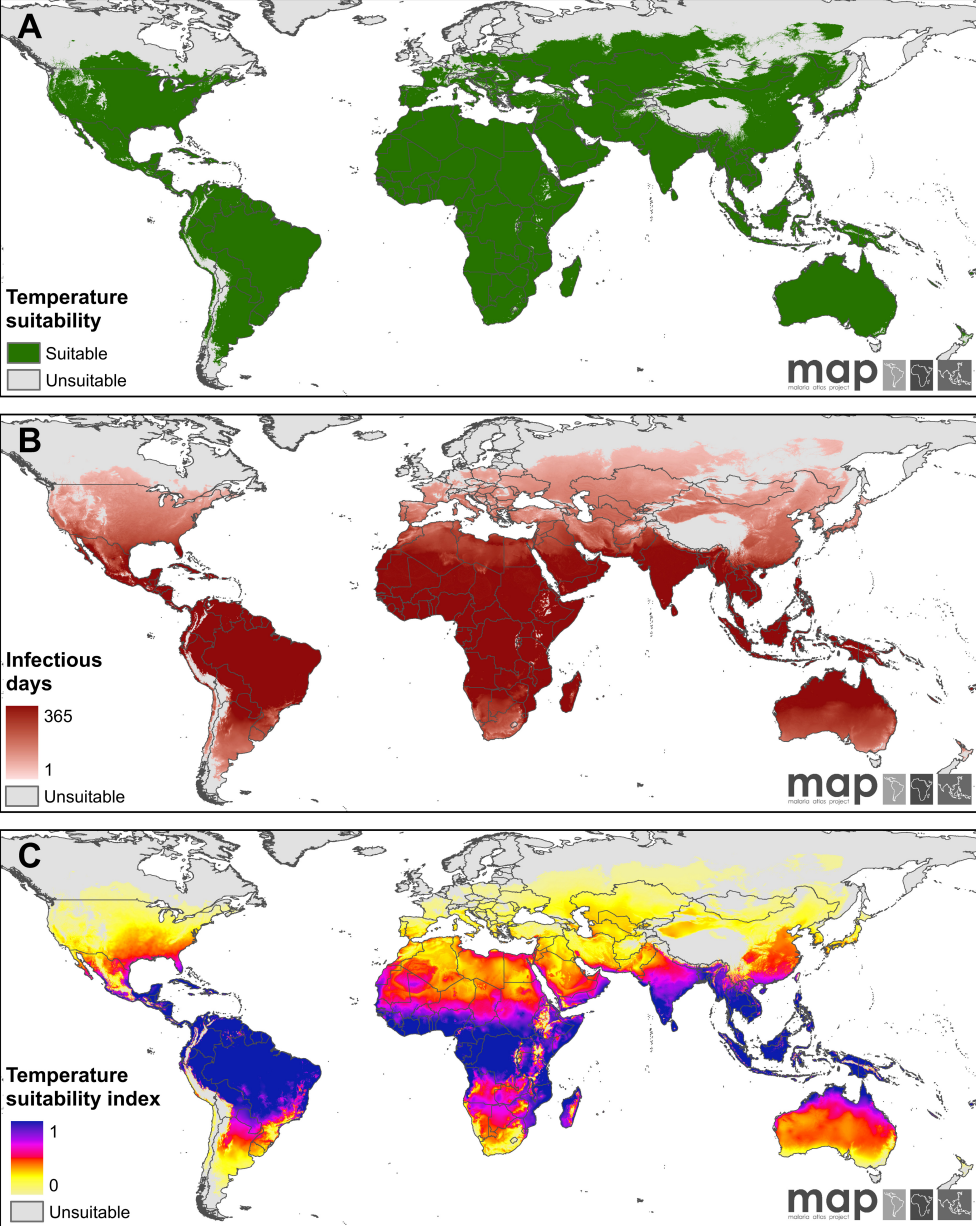
**Mapped outputs of the temperature suitability model for *P. vivax***. Panels correspond directly to those described for *P. falciparum *in Figure 2.

Figures 2 and 3 display global maps of the three indices of temperature-driven suitability for transmission for *P. falciparum *and *P. vivax *, respectively. Figures 2A and 3A map the binary suitability variable for the two parasites: pixels shaded grey are those in which the annual temperature regime precludes the presence of mature sporozoites within local vectors year-round. Conversely, pixels shaded green are able to support sporogony and transmission for some period of time. For both parasites, the northern and southern boundaries of suitability correspond broadly to latitudinal and continental temperature gradients, punctuated by highland and mountainous areas such as the Tibetan plateau, the Andean ridge, and the tropical highlands of East Africa, the Indonesian archipelago and Papua New Guinea. As would be expected, the more rapid parasite development of *P. vivax *means its regions of suitability extend to higher latitudes and encroach further on the highland regions.

Figures 2B and 3B map the estimated number of days in the year for which temperature regimes can support vectors infectious for *P. falciparum *and *P. vivax *, respectively. The areas marked in grey are those in which no days are likely to host infectious vectors, and thus correspond exactly to the 'unsuitable' class in the binary suitability maps described above. For large swathes of the inter-tropical zone, the annual temperature regime can support infectious vectors for 365 days of the year for both parasites. Exceptions to this are the tropical highland regions where the number of days can transition from 365 to zero over relatively short distances. This is especially the case where mountainous regions rise steeply from otherwise low-lying plains, reflecting the abrupt gradients in temperature and, thus, suitability for transmission associated with such topographic features. In contrast to these sharp elevation-driven transitions, far gentler gradations are associated with latitudinal temperature patterns; from year-round suitability in most tropical regions to more seasonal suitability in the sub-tropics and, eventually, very low numbers of suitable days at the latitudinal maximums of the suitable zone. Again, the greater sensitivity of *P. falciparum *to lower temperatures means the number of suitable days for this parasite drops more rapidly with rising latitudes than for *P. vivax*. Globally, an estimated 37.6 million km^2 ^have year-round temperature conditions amenable to transmission for *P. falciparum *(*i.e*. 365 days in which potentially infectious vectors are present) compared to 43.5 million km^2 ^for *P. vivax*.

Figures 2C and 3C map the annual suitability index for *P. falciparum *and *P. vivax *, respectively, derived from the area under the *Z(T) *time-series for each pixel. Like the maps displaying number of infectious days, these illustrate a metric of the degree of temperature suitability for each pixel, but a key difference is the additional contrast displayed by the latter metric. The number-of-days variable can be seen to saturate for large swathes of the tropics; for most of this region, conditions are suitable year-round and thus the variable has no additional capacity to distinguish the extent of suitability. In contrast, the annual suitability index resolves substantial additional variation within the zone of year-round suitability throughout tropical regions of the Americas, Africa, and central and south-east Asia. Comparison of the maps for *P. falciparum *and *P. vivax *, which were standardised to the same scale, illustrates the relatively higher degree of temperature suitability for the latter parasite throughout its range.

Additional Files 3 and 4 are animations displaing the temperature suitability index dynamically through time for *P. falciparum *and *P. vivax *, respectively, presenting in spatio-temporal form the complete *Z(T) *time-series for every 1 × 1 km pixel. Displaying the index in this way allows a rich visualisation of temperature-driven seasonality, with the most striking pattern being the latitudinal advance and retreat of suitability values throughout the year. As would be expected, equatorial regions display the least dynamic variation in temperature suitability, whilst suitability in the high northern and southern latitudes displays marked seasonal oscillation. In the simulated average year, the maximum geographic limits of suitability reach their southern extent during weeks 5-6 (February 4^th ^- 27^th ^) before moving northward as the seasons shift and reaching their maximum northern extent during weeks 31-32 (August 3^rd ^- August 16^th ^). Regional space-time dynamics display a diversity of more subtle patterns around these global scale trends, driven by local temperature regimes. A particularly interesting feature is the effect of extremely high temperatures that prevail across the Sahara, the Arabian peninsula, and into western and central Asia as the northern hemisphere summer progresses. In these regions, warming temperatures initially increase suitability for transmission as sporogony duration decreases but, as high temperatures become extreme, this trend is reversed as the effects of thermal death on vector populations become limiting. Although extremely useful for exploring such dynamics qualitatively, these animations are best considered as visualisations of the underlying space-time "cube" of modelled temperature suitability values, which can be queried to extract time-series for individual pixels or detailed map sequences for any given region and/or time of the year of interest.

## Discussion

We have used a biological model to represent the principal mechanisms by which temperature constrains the transmission of human malaria, and implemented this model in conjunction with high spatial resolution mapped temperature data and a realistic representation of daily and seasonal temperature cycles to describe these effects in fine temporal and spatial detail worldwide for *P. falciparum *and *P. vivax*. The use of a fully dynamic vector lifespan-based approach allows incorporation of the effects of continuously changing temperatures experienced by vector and parasite cohorts and extends previous approaches attempted at the global scale.

We make a number of simplifications in the representation of temperature effects. In particular, we opted to treat the rate of vector emergence - and hence the initial size of each simulated vector cohort - as an arbitrary constant. Our approach has been to capture the limiting or enabling effects of temperature for supporting parasite development within temperature-modulated vector life-spans, and thus the potential for vector populations to include infectious individuals. We make no attempt to model changes in the absolute size of those vector populations through time, and correspondingly the suitability index is relative rather than absolute. We also focus on long-term average temperature regimes and also note that the input temperature data are inevitably subject to uncertainty and local error. As such we do not attempt to predict the implications for transmission suitability of within or between-year deviation from these averages temperatures [61,62], nor of potential long-term changes under climate change [63]. Where actual long-term average temperatures deviate substantially from those suggested by the input data, or where variation around these averages are large, the corresponding values predicted by the index would also change substantially. By focusing on ambient temperatures, we also exclude consideration of the moderating effects of mosquito behaviours and the ability of some species to seek warmer microclimates such as those offered by human dwellings [64].

The modelled output does not, and is not intended to, represent a metric of contemporary malaria endemicity and this disconnect arises for a number of reasons. Firstly, in many regions of the world temperature is not the primary climatic limitation on transmission. In semi-arid or arid regions, in particular, the unavailability of sufficient moisture to provide breeding habitats and prevent desiccation precludes or severely limits transmission regardless of the suitability of ambient temperature regimes [65,66]. This is apparent in regions such as the Sahara, and interior areas of the Arabian peninsula and Australia which are modelled as having suitable temperature regimes but where aridity would prevent transmission. Secondly, climatic suitability models cannot capture factors such as differences in transmission efficiency of locally dominant vector species [67-69] - which largely explains why the Amazonia region is associated with lower levels of endemicity than equivalent equatorial regions of central Africa despite a higher modelled level of temperature suitability; or human susceptibility moderated by inherited blood disorders [70] - which largely explains the known low risk of *P. vivax *transmission to indigenous African populations despite extremely favourable temperature conditions across much of the continent. Thirdly, the impact of human activities - whether deliberate control or more indirect effects arising from socioeconomic development and urbanisation - have had a dramatic effect on the global malaria landscape over the past century [71] and this accounts for the most striking disparities between temperature-defined suitability in large swathes of higher latitude regions of North America, Europe, and Asia, and the absence of endemic transmission for both parasites today [4].

The outputs presented here have a number of direct uses to support malaria public health cartography. Firstly, the mapped limits define the maximum geographic extent of temperature suitability, allowing a fine scale exclusion of regions, and thus populations, not experiencing transmission under average conditions. To our knowledge, these maps provide the first such demarcation for *P. vivax *[6] and an update of an earlier, more rudimentary, attempt for *P. falciparum *[5]. The dynamic visualisations extend this utility from a static exclusion of risk to a temporal one with periods of the year unsuitable for transmission described at each location.

Beyond the binary classification of suitable and unsuitable environments, the temperature suitability index has two distinct uses in malaria cartography. Firstly, because the suitability index is defined within the formal framework of the basic reproductive number, its global and dynamic evaluation at fine spatial resolution represents a resource that can support a wide range of subsequent biological modelling studies needed to support control and elimination efforts at global, regional, or local scales [72]. Secondly, the index can be used as an empirical covariate of malaria endemicity in high spatial resolution geostatistical mapping projects, replacing the ubiquitous direct use of temperature data [4,13-23]. In this role, the index can be thought of as a biological transform of raw temperature data into a mapped variable that acts more directly on, and hence is likely to be more empirically informative of, malaria prevalence, and that can therefore enhance the fidelity of predicted maps. The availability of the index in fine temporal, as well as spatial, resolution also raises the possibility of its more elaborate use as a space-time covariate, extending what are currently nascent developments in empirical malaria modelling [23].

The outputs of this study provide new dynamic cartographic products to inform malaria public health decision making, as well as a resource to support subsequent applied biological and empirical modelling ventures. All outputs are freely available via the Malaria Atlas Project website http://www.map.ox.ac.uk or via direct contact with the authors.

## Competing interests

The authors declare that they have no competing interests.

## Authors' contributions

PWG, SIH, and DLS conceived the analysis and PWG wrote the first draft of the manuscript. PWG, DLS, and TPVB implemented the experiments and generated the model output. APP, CAG, RWS, and SIH contributed to refining the experiments. All authors contributed to the final version of the manuscript.

## Supplementary Material

Additional file 1 **Defining long-term average annual temperature regimes**. Click here for file

Additional file 2 **Computational implementation**. Click here for file

Additional file 3 **Seasonal temperature suitability for transmission of *P. falciparum *(movie)**. The *Z(T) *normalized index of temperature suitability for *P. falciparum *displayed by week across an average year. See text for a full explanation of this metric. Click here for file

Additional file 4 **Seasonal temperature suitability for transmission of *P. vivax *(movie)**. The *Z(T) *normalized index of temperature suitability for *P. vivax *displayed by week across an average year. See text for a full explanation of this metric. Click here for file
