# Supplementary material for: Modelling the global constraints of temperature on transmission of Plasmodium falciparum and P. vivax
Source: Parasit Vectors. 2011 May 26;4:92. doi: 10.1186/1756-3305-4-92 (PMC3115897; doi:10.1186/1756-3305-4-92)
Supplement: Additional file 1 — Defining long-term average annual temperature regimes. [file 1756-3305-4-92-S1.PDF]

**Additional file 1: Defining long-term average annual temperature regimes**

Temperature data were obtained from the WorldClim data resource (http://www.worldclim.org, version 1.4) in the form of climatology surfaces [1] detailing monthly mean, maximum and minimum temperature values at 30-arcsec spatial resolution (corresponding approximately to pixels of 1 × 1 km at the equator). These interpolated climatologies reflect the long-term mean values for the 1950-2000 period and are available globally for each synoptic month, providing a total of 36 surfaces (mean, maximum, and minimum surfaces for each of the twelve months of the year). The use of long-term average data was preferred to land surface temperature time-series obtained, for example, by Earth observation sensors [2,3]. Although the latter potentially provides a richer class of input data capturing variation at multiple temporal scales, they are prone to erroneous or missing values due to cloud error [4]. Additionally, the objective in the current study was to evaluate the suitability for transmission of long-term average conditions and we do not seek here to incorporate the additional complexity introduced by short term temperature variability or inter-annual cycles.

The twelve monthly values for each temperature variable (mean, minimum, maximum) were interpolated temporally for each pixel using cubic splines [5,6] to replace the “staircase” time-series with a smooth representation of changing monthly conditions throughout the year (Figure A1.1A). In addition to seasonal temperature changes, incorporating diurnal cycles has also been shown to be important when considering effects on transmission suitability [7] and complex models have been defined to represent these cycles that incorporate parameters such as times of sunrise and sunset [8,9]. The high parameterisation of these models, and the

difficulties in obtaining the required input data globally, meant that a simpler alternative was implemented in this study. The spline smoothed minimum and maximum temperature curves were interpreted as defining the peak and trough value of each diurnal cycle, and a sinusoidal term was used to represent diurnal variation across this amplitude, with a wavelength of 1 day, and a phase fixed so that minimum and maximum temperatures occurred at midnight and noon respectively (Figure A1.1B). The temperature model was thus defined as:

$$T(t) = \frac{1}{2}\left(T_{\max}(t) - T_{\min}(t)\right)\sin\left(\omega t + \varphi\right) + T_{\text{mean}}(t)$$

Where $T_{\max}$, $T_{\min}$ and $T_{mean}$ are the values of the splined maximum, minimum and mean temperature time-series, respectively; $\omega$ is the angular speed, which was set to $\pi$/0.5 corresponding to a wavelength of 1 day; $t$ is time in days; and $\varphi$ is the phase, which was set to zero to invoke a noon diurnal peak.

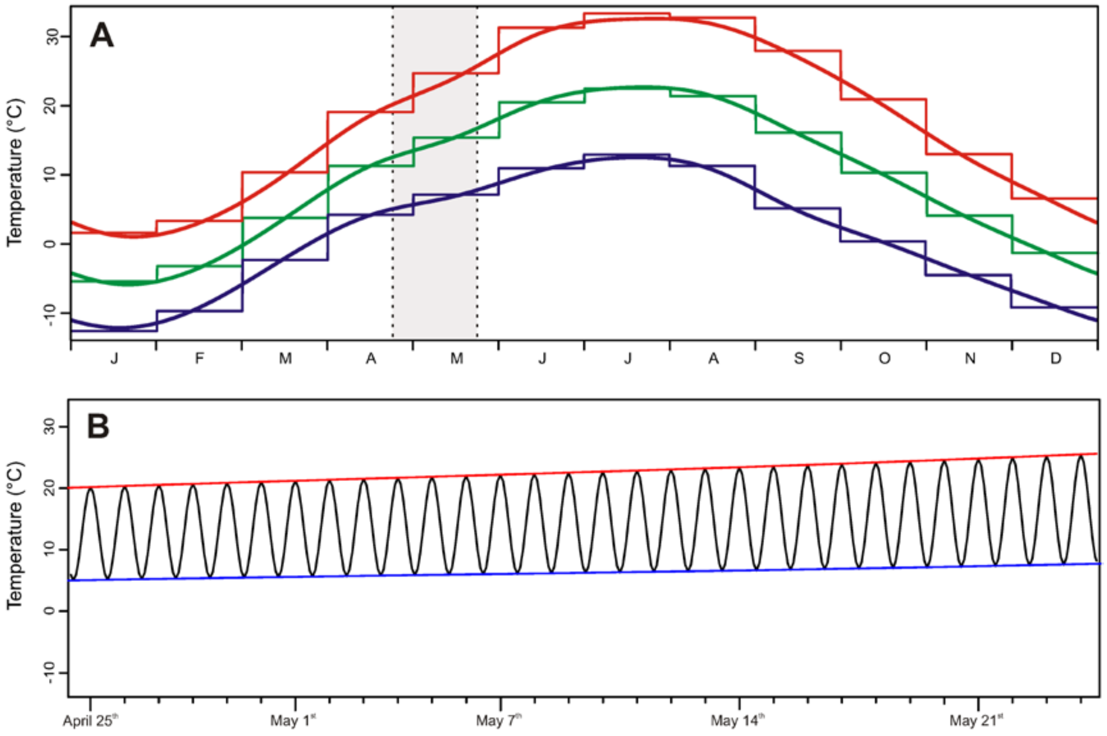


**Figure A1.1 - Modelling annual temperature regimes from monthly mean values.** (A) Blue, green, and red staircase lines represent respectively the raw monthly minimum, mean, and maximum WorldClim temperature climatology data for one example pixel. The corresponding smooth lines were derived from these raw data via cubic spline interpolation. (B) This panel represents a close-up version of the period marked by the grey box in panel A and shows the actual temperature profile (black line) used for modelling generated as a diurnally-varying sine wave spanning the maximum (red line) and minimum (blue line) smoothed time series.

## References


1. Hijmans RJ, Cameron SE, Parra JL, Jones PG, Jarvis A (2005) Very high resolution interpolated climate surfaces for global land areas. Int J Climatology 25: 1965-1978.

2. Tatem AJ, Goetz SJ, Hay SI (2004) Terra and Aqua: new data for epidemiology and public health. Int J Appl Earth Obs 6: 33-46.

3. Hay SI, Tatem AJ, Graham AJ, Goetz SJ, Rogers DJ (2006) Global environmental data for mapping infectious disease distribution. Adv Parasitol 62: 37-77.

4. Scharlemann JPW, Benz D, Hay SI, Purse BV, Tatem AJ et al. (2008) Global data for ecology and epidemiology: a novel algorithm for temporal Fourier processing MODIS data. PLoS One 3: e1408.

5. Becker RA, Chambers JM, Wilks AR (1988) The New S Language. Pacific Grove, CA, USA: Wadsworth & Brooks/Cole.

6. R Development Core Team (2009) R: a language and environment for statistical computing. Vienna, Austria: R Foundation for Statistical Computing, URL: http://www.R-project.org.

7. Paaijmans KP, Blanford S, Bell AS, Blanford JI, Read AF et al. (2010) Influence of climate on malaria transmission depends on daily temperature variation. Proc Nat Acad Sci USA 107: 15135-15139.

8. Paaijmans KP, Read AF, Thomas MB (2009) Understanding the link between malaria risk and climate. P Natl Acad Sci USA 106: 13844-13849.

9. Jetten TH, Martens WJM, Takken W (1996) Model simulations to estimate malaria risk under climate change. J Med Entomol 33: 361-371.
