# Supplementary material for: Modelling the global constraints of temperature on transmission of Plasmodium falciparum and P. vivax
Source: Parasit Vectors. 2011 May 26;4:92. doi: 10.1186/1756-3305-4-92 (PMC3115897; doi:10.1186/1756-3305-4-92)
Supplement: Additional file 2 — Computational implementation. [file 1756-3305-4-92-S2.PDF]

Additional file 2: Computational implementation

The temperature suitability index $Z(T)$ defined in equation (10) of the main manuscript was evaluated discretely using a system of difference equations. Time across the year was discretised into two hour intervals and indexed in units of decimal days since the onset of the year, $0 < t_i \leq 365$. Within each pixel, temperature at each time point, T_i, was computed using the model described in Protocol S1. The probability that a mosquito survives a given interval as a function of temperature during that interval, $p_i(T_i)$, was computed for all time points across the year using the model presented in equation (4) of the main manuscript, thus:

$$p_i(T_i) = \exp\left(\frac{-1}{-4.4 + 1.31T_i - 0.03T_i^2}\right) \qquad \text{(S2.1)}$$

Similarly, the number of degree days available towards completion of sporogony during each interval, $d_i(T_i)$, was computed for each interval across the year based on equation (5) of the main manuscript:

$$d_i(T_i) = (T_i - T_{\min})/12 \qquad \text{(S2.2)}$$

We then defined three relative indices of the vector population: the total number, M; the number infected but not yet infectious (*i.e.* preceding completion of sporogony), Y; and the number infectious (*i.e.* following sporogony), Z, with the latter representing the target value of interest.

Under the condition of a constant daily recruitment rate, λ, we modelled a fixed number of newly emerged vectors adding to the population at each time interval. Since vector populations sizes were modelled in relative, rather than absolute, terms, we choose a convenient arbitrary constant of one additional vector per time step, (*i.e.* λ=12 per day), and then modelled the total relative population of vectors at time point $i+1$ as dependent on the sum of this new recruitment and those surviving from the previous interval as a function of temperature:

$$M_{i+1} = 1 + M_i \cdot p_i(T_i) \qquad \text{(S2.3)}$$

The number of vectors infected but not yet infectious at any point in time, Y_i, was tracked by modelling j=1,2,..., J distinct cohorts; with a new cohort becoming infected at each successive time step. We indexed time for each cohort from the point of infection τ=0 to their maximum lifespan (set at τ = 30 days or τ = 60 days, depending on the longevity parameters of the locally dominant vector species, as described in the main text). The vector population available to become newly infected at each time interval was proportional to M, thus each cohort began its infectious period with a relative population size $Y_{j,\tau=0} = M_i$, and declined in size through time as a function of temperature-dependent survival rates using (S2.1). The progression towards sporogony within each infected cohort was modelled as a function of temperature at each time interval using (S2.2):

$$S_{j,i+1} = S_{j,i} + d_i(T_i) \qquad \text{(S2.4)}$$

and sporogony was considered complete for that cohort when $S_{j,i} \geq \phi$ degree-days (where ϕ=105 and ϕ=111 for *P. vivax* and *P. falciparum* respectively), captured in an indicator variable I:

$$I_{j,i} = \begin{cases} 0 & if \quad S_{j,i+1} \geq \phi \\ 1 & if \quad S_{j,i+1} < \phi \end{cases} \tag{S2.5}$$

The change in population size at each time step of each infected but not infectious cohort was therefore modelled as:

$$Y_{j,i+1} = Y_{j,i} \cdot p_i(T_i) \cdot I_{j,i} \tag{S2.6}$$

such that the population declined through time and was set to zero if sporogony had been achieved.

The index Z_i tracked the population-wide reservoir of infectious vectors at each time interval. Z_i was thus augmented at each time step by the surviving population of any vector cohorts that had newly completed sporogony, and depleted by population loss as a function of temperature:

$$Z_{i+1} = Z_i \cdot p_i(T_i) + \sum_{j=1}^{J} \left[Y_{j,i} \cdot p_i(T_i) \cdot \left(1 - I_{j,i}\right) \right] \tag{S2.7}$$

The algorithms were initialised by setting M and Z to zero, and were run for two full years with the first year discarded to allow for a burn-in period, although visual examination of diagnostic time series indicated that the required period was generally much shorter than a full year. The core numerical code was written in Fortran 90 [1], with input/output handled in R [2]. Global computation at 1 × 1km required the algorithms to be implemented for 221.3 million land pixels, and was completed for each species in around 72 hrs running in parallel across seven Intel Xeon 3.20GHz cores sharing 32 Gb of memory.

References

1. American National Standards Institute (1991) ANSI X3.198-1992 (R1997) / ISO/IEC 1539:1991. American National Standard - Programming Language Fortran Extended.

2. R Development Core Team (2009) R: a language and environment for statistical computing. Vienna, Austria: R Foundation for Statistical Computing, URL: http://www.R-project.org.
